# High Survival of *Lasius niger* during Summer Flooding in a European Grassland

**DOI:** 10.1371/journal.pone.0152777

**Published:** 2016-11-16

**Authors:** Lionel R. Hertzog, Anne Ebeling, Sebastian T. Meyer, Nico Eisenhauer, Christine Fischer, Anke Hildebrandt, Cameron Wagg, Wolfgang W. Weisser

**Affiliations:** 1 Terrestrial Ecology Research Group, Department of Ecology and Ecosystem Management, School of Life Sciences Weihenstephan, Technische Universität München, 85354 Freising, Germany; 2 Institute of Ecology, Friedrich-Schiller-University Jena, Dornburger Str. 159, 07745 Jena, Germany; 3 German Centre for Integrative Biodiversity Research (iDiv) Halle-Jena-Leipzig, Deutscher Platz 5e, 04103 Leipzig, Germany; 4 Institute of Biology, University of Leipzig, Johannisallee 21, 04103 Leipzig, Germany; 5 Institute of Geoscience, Friedrich-Schiller-University Jena, Burgweg 11, 07749 Jena, Germany; 6 Max Planck Institute for Biogeochemistry, POB 100164, 07701 Jena, Germany; 7 Department of Evolutionary Biology and Environmental Studies, University of Zürich, Winterthurestrasse 190, CH-8057, Zürich, Switzerland; Estacion Experimental de Zonas Áridas (CSIC), SPAIN

## Abstract

Climate change is projected to increase the frequency of extreme events, such as flooding and droughts, which are anticipated to have negative effects on the biodiversity of primary producers and consequently the associated consumer communities. Here we assessed the effects of an extreme early summer flooding event in 2013 on ant colonies along an experimental gradient of plant species richness in a temperate grassland. We tested the effects of flood duration, plant species richness, plant cover, soil temperature, and soil porosity on ant occurrence and abundance. We found that the ant community was dominated by *Lasius niger*, whose presence and abundance after the flood was not significantly affected by any of the tested variables, including plant species richness. We found the same level of occupation by *L. niger* at the field site after the flood (surveyed in 2013) as before the flood (surveyed in 2006). Thus, there were no negative effects of the flood on the presence of *L. niger* in the plots. We can exclude recolonisation as a possible explanation of ant presence in the field site due to the short time period between the end of the flood and survey as well as to the absence of a spatial pattern in the occupancy data. Thus, the omnipresence of this dominant ant species 1 month after the flood indicates that the colonies were able to survive a 3-week summer flood. The observed ant species proved to be flood resistant despite experiencing such extreme climatic events very rarely.

## Introduction

**Fig 1:**
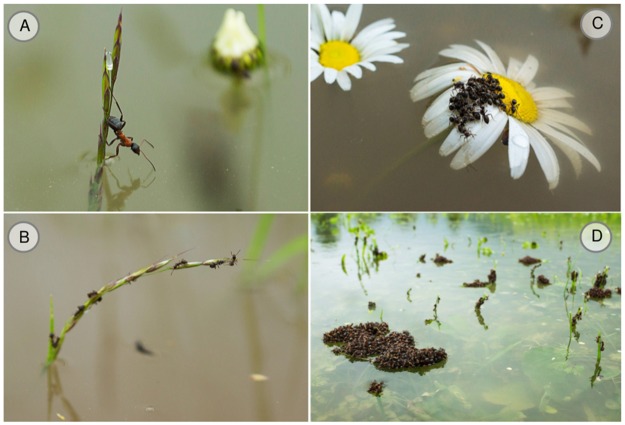
Impressions from the flood. A: Ant worker clinging to the tip of a grass blade. B: Several ant workers clinging to the tip of the grass blade. C: Group of ant workers on a Bellis perennis flower. D: Ant rafts.

One of the predicted consequences of climate change is an increase in the frequency of extreme rainfall events and flooding in many catchments worldwide [1]. A direct effect of flooding is the destruction of primary productivity, faunal habitat as well as the displacement of local flora and fauna to new locations. Such strong ecosystem perturbations may function to reset the affected ecosystem [2]. More specifically, many arthropods are particularly susceptible to the impact of flooding because part of their life-cycle is spent below ground and have low mobility [3]. For some arthropods, the inability to escape extreme flooding events may have ecological and evolutionary consequences on the survival or recolonisation strategies of arthropod individuals after flooding events. In habitats where cyclical or frequent flooding occurs, for instance in the central Amazonian river floodplains, numerous life-histories or behavioural strategies that allow arthropod individuals either to survive flooding or to quickly re-colonise recently flooded habitats have been described [4]. High tolerance of anoxic conditions has been observed in ants [5]; however, behavioural strategies for surviving major flood events can be extremely diverse. For example, worker or soldier ants may attempt to preserve the colony by plugging the nest entrances with their heads [6, 7], or with loose soil particles [8] and may even excrete excess water outside the nest through communal urination [6]. Ants may additionally attempt to preserve the colony through escape by the formation of worker rafts [9] (Fig 1) or even by walking on water [5]. However, most of these adaptations are reported in tropical habitats, where regular flood events lead to strong selective pressures to evolve adaptive strategies. In contrast, for ants occupying central European temperate habitats that are not frequently or intensely flooded, such as grasslands, adaptations to survive floods on-site have not been described. Flood avoidance by moving upland or raft formation combined with a rapid recolonisation of the recently disturbed habitat has been the most described strategy [10]. Therefore, for colonial species, such as ants, a major trait for successful recolonisation is the ability to build new nests rapidly [11]. Alternatively, survival of ants to flooding of the nests might be assisted by their high tolerance to such hypoxic conditions arising within their nest under normal conditions [12]. Some authors already reported high flooding tolerance of temperate species to short flooding [13], therefore temperate ants may be predisposed to survive infrequent, short flooding [14]

Negative impacts of major flooding events on an ant colony may additionally be mitigated by the habitat in which colonies are established. For instance, the impact of flooding on a temperate ant community may be mediated by local plant diversity. Previous theoretical [15] and empirical [16] models have revealed a positive relationship between the diversity and stability of ecological communities, such as the abundance of arthropod populations [17]. Moreover, higher densities of ant nests have been shown to occur in plots with higher plant species richness (Clemens et al. unpublished). If habitats more diverse in plant species are preferred for colony establishment, this may indicate that the surrounding habitat can provide resources and security from predators and environmental perturbations. This preference could lead to a greater chance of finding ants in diverse habitats. There are various possible mechanisms explaining how plant diversity could affect the survival of ants. Firstly, plant species richness increases soil porosity [18], which may reflect on the availability of oxygen in the soil and hence promote survival of ants in waterlogged soils [19]. Second, in temperate grasslands, plant cover and vegetation height increase with plant species richness [20]. Higher and denser plant patches, in turn, provide larger numbers of temporary refuges (e.g. tip of the vegetation and flower heads) which could enhance the survival of individuals (Fig 1).

Natural flooding events provide an opportunity for observational studies that are required to evaluate if and how temperate ant species can survive flooding, and demonstrate any specialised adaptations, as has been observed in their tropical relatives. In central Europe during May 2013, such an opportunity arose when temperate European ecosystems, particularly river basins, experienced a severe flooding event resulting in the submergence of grassland communities for an extended period, including the long-term biodiversity experiment in Jena, Germany [21]. Here we investigated the 2013 flood impact on ant populations within an experimental field site where plots within the site varied in plant diversity [22]. During the initial few days of the flood, we observed high arthropod mortality, and many dead and dying insects appeared in the water. Similar to other insects, we observed ant workers clinging to the tips of the vegetation (Fig 1). The experimental plots were submerged for a period between 4 and 24 days, and after approximately 3 days, we could no longer observe living arthropods at the field site. Because of the magnitude and timing of the flood, we did not expect a high survival of ant colonies on-site. However, within the first few days of the flood retreating, we observed ant workers tending aphids that probably recolonised the site after the flood due to their high dispersal abilities. These unexpected observations led us to investigate potential causes explaining the survival of ant colonies at the field site. The following hypotheses were formed in this study: 1) the longer a given location remained submerged during the flood, the lower the occupancy and abundance of ants and; 2) the local plant diversity mitigated the negative impacts of flooding on ants; therefore, we can expect the occupancy and abundance of ants to be greater in habitats with more diverse plant species. We discuss our results and potential mechanisms that allow ant colonies to survive, rather than recolonise a field site after a flood. As a control comparison to ant occupancy before the major flooding event, we compared our data with a previous survey conducted during 2006.

## Materials and Methods

### Ethics statement

Plant and arthropod sampling were conducted with the permission of the city council of Jena, Germany.

### Experimental field site

**Fig 2:**
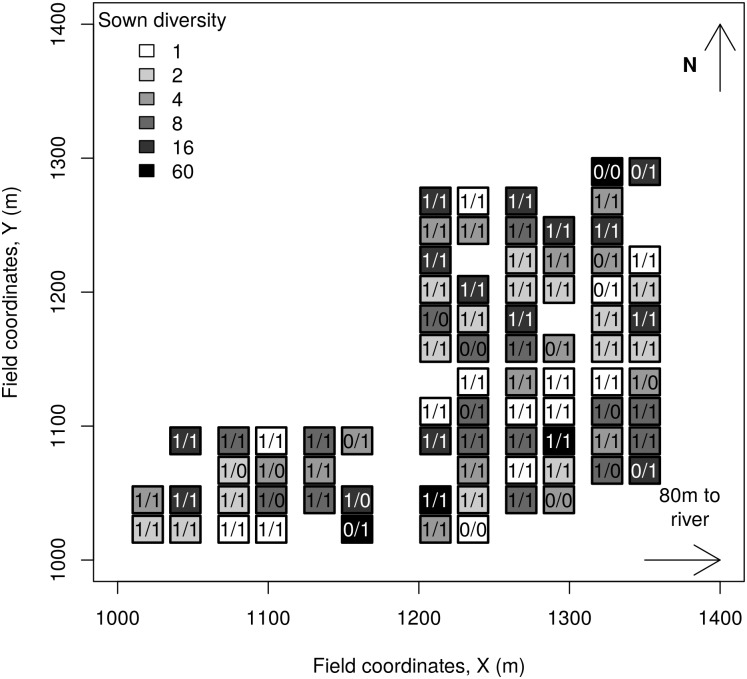
Sketch of the field site of the Jena Experiment. Each rectangle represents a plot. The shades of grey indicate the various levels of plant species richness. The number within each plot represents the presence (1) or absence (0) of ants at the respective plot. The number on the left of the slash represents data from the survey conducted in 2006, whereas the number on the right represents the data from the survey conducted in 2013. The Saale River runs parallel to the field site approximately 80 m to the eastern edge.

The survey was conducted at the field site of the Jena Experiment (Thuringia, Germany, 50°55´ N, 11°35´ E, 130 m above sea level [22]), a former arable land located in the floodplain of the Saale river. Mean annual air temperature and precipitation from 1961 to 1990 were 9.3°C and 587 mm [23], respectively. The experiment was established in 2002 and consist of 80 plots of 20 x 20 m sown with a defined number of grassland plant species originating from a pool of 60 species and forming a gradient with six levels of sown plant species richness (1, 2, 4, 8, 16 and 60 species). The plant species are all commonly found in Molinio-Arrhenatheretea meadows [24]. To account for the change of soil texture with distance from the Saale river, four blocks were established parallel to the river. The plots were arranged in two rows in blocks 1–3 and in 6 rows in block 4 (Fig 2). Plots in block 4 were the farthest from the river, whereas plots in block 1 were the closest. The elevation of the experimental plots does not vary by more than 2m and is not correlated with the distance to the Saale river. A reference point was set at the south-west corner of the field site, and distances from this point to the centre of the plots were measured in both a west-east gradient (X) and a south-north gradient (Y). The sizes of the plots were reduced to 6 x 7 m in 2009. The experimental plots are manually weeded three times per year and mown twice per year. The distance between the plots of the experiment and the Saale river ranges from more than 400 m to approximately 80 m.

### Flooding event

The Saale floodplain in Jena has rarely been flooded since the installation of a hydroelectric dam regulating water levels. Since the establishment of the experiment during 2002, two such events have occurred during the late winters of 2003 and 2011. However, the flood during the summer of 2013 was unique in that it occurred during the period of peak biomass and high biotic activity. During May and June 2013, many regions of central Europe experienced heavy and frequent rainfall. Extensive regions in central Germany received up to 300% of the long-term average (1961–1990) rainfall in May [25], which together with already wet antecedent conditions [26] cumulated into an extreme flooding event [27] that included the Saale river in central-eastern Germany. Within the first day of the flood (31st May 2013), all plots within the experimental field site as well as all adjacent fields were submerged. The closest area from the experimental plots not submerged on this day was approximately 50 m to the west of the field site. Thus, if post-flood recolonisation were to occur, it would likely have originated from this direction (Fig 2). From that day onwards, the water retreated until June 24th 2013, when plots were no longer submerged. During the flood event, on a daily basis, we noted whether plots were submerged [21]. As a proxy of flood duration, we used the sum of days for which plots were submerged. The plot-specific flood duration ranged from 4 to 24 days.

### Measurement of soil properties

To account for the spatial variation of soil properties, three replicates were taken per plot, using sample rings with an internal diameter of 57 mm and height of 40.5 mm (inner volume of 100 cm^3^) approximately 2, 3 and 4 weeks after the flood during June 2013. The samples were saturated with water and then dried at 105°C and weighed to calculate soil porosity (%) (0–5 cm soil depth) and the average soil porosity value was used in subsequent analyses. The soil temperature (in °C) was determined using a frequency domain sensor probe (Wet-2 Sensor, Delta-T Devices, Cambridge, United Kingdom) on 1st August 2013. The device was inserted 6 cm deep (length of the prongs) into the soil from the top, and the average of three measurements was used in the analysis. During 2006, a PT100 resistance thermometer was inserted 5 cm into the ground, and the temperature was recorded every hour of the day during July 2006, although we used the monthly average value in the analysis. Soil temperature was included in the analysis to control for its effect on activity rates of ant workers.

### Plant community measurements

We used the plant cover data collected during May 2013 to assess the impact of cover at the time of the onset of the flood on ant survival. As higher plant cover could provide temporal refugia for ant individuals, we used the cover data collected just before the flood in our analysis. For the 2006 data, we used plant cover values computed during August close to the time of sampling. To quantify the cover of each of the plant species, we used a decimal scale within a predefined 3 x 3 m area of the main experiment [28].

### Ant survey

The ant community in the study site is largely dominated by *Lasius niger* (Linneaus, 1758). A previous sampling campaign in 2005 revealed that this species accounted for 94% of all collected ant workers at the site (Voigt et al. unpublished). The other common ant species at the site is *Myrmica scabrinodis* (Nylander, 1958), which made up 4% of collected ant individuals. All other ant species showed proportional abundances lower than 1%. Ant occurrence was measured in the experimental plots before (summer 2006) and after the flood (summer 2013). For both surveys, we used two different kinds of baits (tuna and sugar in 2006 and tuna and honey in 2013) to attract a wide range of different ant species. Honey baits have been shown to attract higher numbers of ants than those with sugar baits [29]; therefore, we expected to find a higher number of ants in the 2013 sample. This effect prevented us from directly comparing the activity data between the two years. Nevertheless, baits side effects are equal across the plots within each year, which still enables us to look at the effect of plot-level variables on ants within each year. The ant survey during 2006 was conducted on 24th July 2006 and from July 30th until 3rd August during 2013, 36 days after the end of the flood. Except for the difference in the carbohydrate-rich bait (sugar vs. honey), the same methods were used during both years. Approximately 10 g of bait material was placed on the soil surface in two different petri dishes of 20-cm diameter. These were located at the same position within each plot. All handling was performed with rubber gloves. Thirty minutes after locating the petri dishes on the plots, the number of ant workers present in the petri dish was assessed. During the 2013 sampling one ant individual was selected at random from each plot and preserved in 70% ethanol for later identification. Due to handling issues, no ant individuals could be collected from 10 plots, even through the number of ant individuals was recorded. The collected ant individuals were identified using the identification keys by Seifert [30].

### Statistical analyses

We tested the variation in ant occurrences and abundances in response to plot-level characteristics. These responses were measured at the plot level. Ants may colonise the field through the south, west and north margins, and since the east margin is close to the river and consists of a forested strip, we did not consider it as a potential source of ants. This means that under a recolonisation scenario, we expect a negative slope between the plot X coordinates and ant occurrence. Because colonisation may originate from both the south and north margins, we included a quadratic Y term in the models. Again, under a recolonisation scenario, this quadratic term should be positive. The tested explanatory variables were: X coordinates of the centre of the plots, Y coordinates of the centre of the plots, square of the Y coordinates of the centre of the plots, flood duration (days), soil porosity, plant cover, plant sown richness and interaction between the plant sown richness and the flood duration. Plant sown richness was log-transformed as we expect this variable to affect survival of ants asymptotically; the log-transformation ensures that this relationship is then linear. We additionally expected that plant richness may buffer ant colonies against flooding effects [21] and included an interaction term between these two variables in the models. We did not expect other interactions, such as soil variables and plant cover should affect ant colonies independent of plant richness. The spatial blocks in which plots are arranged was not included in the model as it is inherently incorporated in the model by the plot coordinate parameters. We used generalised linear models with a binomial distribution (logit link) for the ant presence–absence, we also checked for overdispersion but could not find any indication of it in our data. For the ant abundance data we used a negative binomial distribution (log link). The significance of the explanatory variables was assessed using a sequential analysis of deviance where the drop in deviance after the addition of each variable was assessed using a chi-square test. The ordering of the variables was based on a priori knowledge as we wanted to control for plot position before testing flood effects, soil variables and plant cover. Plant richness was included as the last variable so that we may identify if a significant amount of left-over deviance, not explained by any of the previous variables, is explained by plant richness. The data from 2006 and 2013 were separately modelled. Pseudo R^2^ values for the models were computed using the McFadden equation [31]. All statistical analyses were conducted using R version 3.02 [32].

## Results

During 2006, ants were found in 68 out of 80 (85%) plots during the baiting survey. During 2013, approximately 1 month after the flood, the proportion of plots occupied by ants was the same as that during 2006 (Fig 2). The number of individuals found in the plots ranged from 0 to 67, with an average of 10 individuals, in 2006 and from 0 to 212, with an average of 38 individuals, during 2013. The abundances were higher in 2013 than in 2006 (Wilcoxon sign-rank test, V = 2309, p-value< 0.001). Of the 58 individuals collected during 2013, 57 were *L. niger* and the single other individual was *M. scabrinodis*.

### Ant occurrence and plot properties

Similar patterns between 2006 and 2013 were evident from the average values of variables in plots where ants were either absent or present (Table 1). For the data obtained from the survey conducted in 2006, only the Y-coordinates of the plots had a significant positive coefficient, whereas during 2013 only the quadratic term for the Y-coordinates of the plot led to a significant drop in mode deviance (Table 2). The pseudo-R^2^ for the 2013 model was 0.15, whereas it was 0.10 for the 2006 model.

**Table 1:** Average variables values with standard deviation in parenthesis in plots for 2006 and 2013 where ants were absent or present.

Variable	2006		2013	
	Absence	Presence	Absence	Presence
Flood	-	-	14.41 (4.07)	14.23 (4.90)
Soil porosity	-	-	51.20 (3.64)	49.58 (2.76)
Soil temperature	17.54 (0.52)	17.73 (0.65)	14.46 (0.81)	14.56 (0.75)
Plant cover	94.08 (11.74)	81.95 (13.48)	99.21 (20.14)	93.09 (36.28)
Sown richness	10.92 (15.95)	8.40 (12.41)	15.50 (21.36)	7.59 (10.59)

**Table 2:** Ant presence-absence in response to the plot coordinates (Fig 2), log of the sown plant richness, flood index (only 2013), plant cover, soil porosity (only 2013), soil temperature and interaction between the plant richness and the flood index (only 2013). Reported are the model coefficients (with standard error in brackets), the p-values associated with the model coefficients, the drop in residual deviance as each term is sequentially added to the model (Deviance) and the p-values associated with this drop based on a chi-square test. The degrees of freedom used in the chi-square test are depicted in subscripts.

Variable	2006				2013			
	Coefficients	P values	Deviance	P values	Coefficients	P values	Deviance	P values
X	-0.42 (0.39)	0.286	0.06_1_	0.794	-1.07 (0.63)	0.091	2.89_1_	0.082
Y	0.92 (0.45)	0.046	2.29_1_	0.129	0.71 (0.46)	0.122	0.19_1_	0.659
Y^2^	-0.37 (0.42)	0.393	0.51_1_	0.474	-0.66 (0.43)	0.124	4.56_1_	0.032
Flood duration	-	-	-	-	-0.20 (0.46)	0.654	0.17_1_	0.679
Soil porosity	-	-	-	-	-0.30 (0.52)	0.565	1.45_1_	0.227
Soil temperature	-0.12 (0.39)	0.757	0.80_1_	0.369	-0.07 (0.52)	0.898	0.00_1_	0.944
Plant cover	-0.76 (0.45)	0.099	3.10_1_	0.078	-0.23 (0.40)	0.566	0.18_1_	0.667
Sown richness	-0.20 (0.35)	0.561	0.33_1_	0.561	-0.00 (0.51)	0.996	0.18_1_	0.874
Flood:Richness	-	-	-	-	0.43 (0.44)	0.332	0.95_1_	0.327

### Ant abundance and plot properties

During 2013, none of the tested explanatory variables had a significant effect on ant abundance (Table 3). During 2006, soil temperature had a positive effect on ant abundance, plant cover had a negative effect, and the quadratic term for the Y-coordinates of the plot centre was significantly negative. The pseudo-R^2^ values were 0.23 and 0.05 for 2006 and 2013, respectively.

**Table 3:** Ant abundance in response to the plot coordinates (Fig 2), log of the sown plant richness, flood index (only 2013), plant cover, soil porosity (only 2013), soil temperature and interaction between the plant richness and the flood index (only 2013). Reported are the model coefficients (with standard error in brackets), the p-values associated with the model coefficients, the drop in residual deviance as each term is sequentially added to the model (Deviance) and the p-values associated with this drop based on a chi-square test. The degrees of freedom used in the chi-square test are depicted in subscripts.

Variable	2006				2013			
	Coefficients	P values	Deviance	P values	Coefficients	P values	Deviance	P values
X	-0.06 (0.16)	0.705	1.62_1_	0.202	-0.18 (0.27)	0.497	1.42_1_	0.233
Y	0.26 (0.0.18)	0.151	0.15_1_	0.696	0.23 (0.23)	0.303	0.05_1_	0.810
Y^2^	-0.51 (0.16)	0.002	7.92_1_	0.004	-0.39 (0.22)	0.076	1.49_1_	0.222
Flood duration	-	-	-	-	-0.00 (0.18)	0.962	0.00_1_	0.981
Soil porosity	-	-	-	-	0.10 (0.25)	0.690	0.00_1_	0.930
Soil temperature	0.26 (0.16)	0.107	13.77_1_	0.000	0.33 (0.25)	0.176	0.89_1_	0.344
Plant cover	-0.38 (0.16)	0.015	4.57_1_	0.032	-0.04 (0.20)	0.825	0.13_1_	0.719
Sown richness	-0.10 (0.14)	0.482	0.46_1_	0.494	-0.17 (0.25)	0.491	0.21_1_	0.646
Flood:Richness	-	-	-	-	0.19 (0.17)	0.268	0.79_1_	0.372

## Discussion

Here we hypothesised that 1) the longer a local area remained submerged during a flood, the lower was the occupancy and abundance of ants and 2) if the local plant diversity mitigates the negative impact of severe flooding on colonies, we would expect the occupancy and abundance of ants to be greater in habitats more diverse in plant species immediately after the flood had receded. However, we found little support for both our hypotheses since during 2013, only 1 month after an extensive summer flood, ants were found to occur in the same proportion of plots of the Jena Experiment as observed in a previous survey during 2006, which was not affected by flooding. Moreover, the same ant species, *L. niger*, was found to dominate the field site, both before and after the flood. During 2006, an increase in ant abundance with soil temperature as well as a decline in ant abundance with plant cover was detected. Meanwhile, during 2013, none of the tested variables had an effect on ant abundances and only the quadratic term for the Y-coordinates of the plot had an effect on ant occurrences. Thus, there was no detectable effect of the flooding event, even when plots remained submerged for up to 3 weeks.

### Recolonisation or survival?

The ant individuals and colonies observed 1 month after the flood might have re-colonised the field site, thereby filling the disturbance induced empty niche. However, several characteristics of the ant community present at our field site make the rapid colonisation of the field site after the flood unlikely. First, mating flights of *L. niger* occur during late summer until the beginning of autumn [30] and colonisation of new habitats is only possible after that. We are not aware of any reported instance of movement of adult *L. niger* colonies. Second, the observed ant activity (on average 38 individuals observed per plot during 2013) was higher than that expected for young colonies, newly established after the flood [30]. Third, there was no spatial pattern of higher ant occurrence at the edges of the field site that would indicate recolonisation patterns or movement of adult colonies (Fig 2). Moreover, the effects of the plots coordinates on ant occurrences did not support the recolonisation hypothesis. The X-coordinates, which are inversely proportional to the distance to the river, did not affect ant occurrence and abundance. Similarly the Y-coordinates, which represent a south-north gradient, led to an increase in the probability to find ants in 2006 but not in 2013 and had no effects on ant abundance. In addition, the quadratic term of the Y-coordinates was never significantly positive as expected from a recolonisation scenario. The minimum distance between one of the experimental plots and field margins being approximately 50 m and the territory size of *L. niger* being on average 36 m^2^ [33], patrolling ants are then expected to be recorded up to 18 m away from their nests, with declining numbers as the distance to the nest increases [34]. It is then unlikely that the ants detected in the current study originate from ant nests situated outside the field site and not affected by the flood. Therefore, we conclude that *L. niger* colonies have most likely survived the flooding and consequently possess strategies to resist infrequent flooding.

### Potential survival mechanisms

*Lasius niger* is a widely distributed ant species in European grasslands [35]. To date, no specific adaptations of *L. niger* to flooding have been described. Due to the stochastic characteristic of flooding events in this particular habitat, we did not expect strong adaptation pressure for the species, although water logging of the soil without surface inundation may frequently be occurring but is not recorded. Therefore, the question arises as to how the colonies were able to survive the heavy summer flood during June 2013, when our experimental plots were submerged between 4 and 24 days with a mean of 14 days, causing anaerobic soil conditions [21]. Ant individuals could have survived the flood outside their nests. Purcell et al. [36] demonstrated that raft formation by *Formica selysi* (Bondroit, 1918) colonies in a temperate alpine floodplain greatly improved colony recovery after a flood. During the flood, we similarly observed ant rafts in our field site (Fig 1) and many ants clinging to vegetation extending above the water surface. However, we found many ant workers outside their nests only during the first few days of the flood. We assumed that most of these ant individuals were not able to survive outside their nests due to predation or being carried away by the water. Based on our observations, we propose that ants must have survived below the water, most likely inside the colonies, in contrast to the ‘risk strategy’ reviewed by Adis and Junk [4]. Other temperate ant species, such as *M. rubra* (Linneaus, 1758), have already been reported to show high nest resistance to flooding [37]. Under experimental conditions, it has been shown that *L. niger* workers can withstand a prolonged period underwater. For instance, Boomsma and Isaaks [14] showed that when submerged under water with air bubbles, survival of *L. niger* workers drops below 50% only after 9–10 weeks of submersion. Although no important physiological adaptations, such as anaerobiosis, were detected in *L. niger*, Boomsma and Isaaks [14] found that not only workers but also queens were able to sustain prolonged submersion and showed that the production of eggs by *L. niger* queens was only affected after 8 weeks of submersion with no air bubbles. In addition Dietrich [13] reported that ant colonies in an Austrian floodplain appeared undamaged by a flooding lasting between 10 hours and 4 days. Finally a previous meta-analysis of terrestrial invertebrates in grassland showed that survival in flooded conditions ranges from a few days to 270 days across different taxa [38]. Therefore, we conclude that a combination of the high ability of individual ants to survive being submerged underwater and potential adaptations of the colonies to trap air within the nests appear the most likely explanations for the observed high survival of ants during the flood in our study.

### Conclusions

Our findings indicate that the high occupancy of *L. niger* at the experimental site after a major flooding event is probably not due to recolonisation, but rather a high survival of the colonies. *L. niger* colonies at our field site are apparently able to sustain stochastic and prolonged flood events due to their remarkably high resistance to submergence. The resistance of such ubiquitous species to disturbance may provide higher stability in ecosystem functioning under the on-going increasing anthropogenic and climatic pressures on biological systems through their key role as ecosystem engineers. Further studies on the impacts of flooding events on other groups of arthropods are necessary to understand if this resistance extends to other taxa and if plant diversity plays a role in promoting the resistance of arthropod communities and their resilience to such disturbances. Resistance to extreme climatic events is of crucial importance because ongoing global change results in heavy pressure on natural ecosystems.
